# CT-Based Radiomics Analysis to Predict Histopathological Outcomes Following Liver Resection in Colorectal Liver Metastases

**DOI:** 10.3390/cancers14071648

**Published:** 2022-03-24

**Authors:** Vincenza Granata, Roberta Fusco, Sergio Venanzio Setola, Federica De Muzio, Federica Dell’ Aversana, Carmen Cutolo, Lorenzo Faggioni, Vittorio Miele, Francesco Izzo, Antonella Petrillo

**Affiliations:** 1 Division of Radiology, Istituto Nazionale Tumori IRCCS Fondazione Pascale—IRCCS di Napoli, 80131 Naples, Italy; s.setola@istitutotumori.na.it (S.V.S.); a.petrillo@istitutotumori.na.it (A.P.); 2 Medical Oncology Division, Igea SpA, 80013 Napoli, Italy; r.fusco@igeamedical.com; 3 Department of Medicine and Health Sciences “V. Tiberio”, University of Molise, 86100 Campobasso, Italy; demuziofederica@gmail.com; 4 Division of Radiology, Università degli Studi della Campania Luigi Vanvitelli, 80138 Naples, Italy; federica.dellaversana@unicampania.it; 5 Department of Medicine, Surgery and Dentistry, University of Salerno, 84084 Salerno, Italy; carmencutolo@hotmail.it; 6 Department of Translational Research, University of Pisa, 56126 Pisa, Italy; lfaggioni@sirm.org; 7 Division of Radiology, Azienda Ospedaliera Universitaria Careggi, 50134 Florence, Italy; vmiele@sirm.org; 8 Italian Society of Medical and Interventional Radiology (SIRM), SIRM Foundation, 20122 Milan, Italy; 9 Division of Hepatobiliary Surgical Oncology, Istituto Nazionale Tumori IRCCS Fondazione Pascale—IRCCS di Napoli, 80131 Naples, Italy; f.izzo@istitutotumori.na.it

**Keywords:** radiomics analysis, liver metastases, computed tomography, prediction of histopathological outcomes

## Abstract

**Simple Summary:**

The objective of the study was to assess the radiomic features obtained by computed tomography (CT) examination as prognostic biomarkers in patients with colorectal liver metastases, in order to predict histopathological outcomes following liver resection. We obtained good performance considering the single significant textural metric in the identification of the front of tumor growth (expansive versus infiltrative) and tumor budding (high grade versus low grade or absent), in the recognition of mucinous type, and in the detection of recurrences.

**Abstract:**

Purpose: We aimed to assess the efficacy of radiomic features extracted by computed tomography (CT) in predicting histopathological outcomes following liver resection in colorectal liver metastases patients, evaluating recurrence, mutational status, histopathological characteristics (mucinous), and surgical resection margin. Methods: This retrospectively approved study included a training set and an external validation set. The internal training set included 49 patients with a median age of 60 years and 119 liver colorectal metastases. The validation cohort consisted of 28 patients with single liver colorectal metastasis and a median age of 61 years. Radiomic features were extracted using PyRadiomics on CT portal phase. Nonparametric Kruskal–Wallis tests, intraclass correlation, receiver operating characteristic (ROC) analyses, linear regression modeling, and pattern recognition methods (support vector machine (SVM), k-nearest neighbors (KNN), artificial neural network (NNET), and decision tree (DT)) were considered. Results: The median value of intraclass correlation coefficients for the features was 0.92 (range 0.87–0.96). The best performance in discriminating expansive versus infiltrative front of tumor growth was wavelet_HHL_glcm_Imc2, with an accuracy of 79%, a sensitivity of 84%, and a specificity of 67%. The best performance in discriminating expansive versus tumor budding was wavelet_LLL_firstorder_Mean, with an accuracy of 86%, a sensitivity of 91%, and a specificity of 65%. The best performance in differentiating the mucinous type of tumor was original_firstorder_RobustMeanAbsoluteDeviation, with an accuracy of 88%, a sensitivity of 42%, and a specificity of 100%. The best performance in identifying tumor recurrence was the wavelet_HLH_glcm_Idmn, with an accuracy of 85%, a sensitivity of 81%, and a specificity of 88%. The best linear regression model was obtained with the identification of recurrence considering the linear combination of the 16 significant textural metrics (accuracy of 97%, sensitivity of 94%, and specificity of 98%). The best performance for each outcome was reached using KNN as a classifier with an accuracy greater than 86% in the training and validation sets for each classification problem; the best results were obtained with the identification of tumor front growth considering the seven significant textural features (accuracy of 97%, sensitivity of 90%, and specificity of 100%). Conclusions: This study confirmed the capacity of radiomics data to identify several prognostic features that may affect the treatment choice in patients with liver metastases, in order to obtain a more personalized approach.

## 1. Introduction

Using radiomics, it is possible to extract multiple quantitative datasets from medical images that can be indirectly linked to pathophysiological characteristics. Radiomic features, if linked with pertinent outcomes elements, can provide precise, evidence-based clinical-decision support systems (CDSS) [1,2,3,4,5]. The potential of radiomics to increase CDSS is incontestable, and its application is evolving quickly [5,6,7]. The main theory of this approach is based on the idea that quantitative data are more understandable in relation to clinical endpoints than qualitative diagnostic evaluation [8,9]. Radiomic features have significant advantages over qualitative evaluation, and this is evidently correlated with the resolution of observers’ eyes. The main difficulty is finding the correct grouping and combination of quantitative data sources that provide a method that accurately and robustly allows outcome prediction as a function of the impending decisions [10,11,12,13,14]. Radiomic features capture tissue and lesion characteristics, such as heterogeneity and shape, and can be used to assess tissue heterogeneity, either alone or in combination with prognostic data. Several studies have shown that radiomic characteristics are strongly correlated with heterogeneity indices at the cellular level [1,2,3,4,5,6,7,8]. Radiomics can support cancer detection, diagnosis, prognosis assessment, and response to treatment, as well as supervise disease status [3].

Recently, there has been a significant increase in radiomics investigations, including in the liver field: liver fibrosis assessment, characterization of malignant and benign lesions, and prognosis [15,16,17,18,19,20,21].

Colorectal carcinoma (CRC) is the cancer with the third-highest incidence and second-highest mortality rate [22]. The most common site of metastases is the liver, with 75–80% of patients unfit for curative surgical resection of colorectal liver metastases (CRLMs) [23]. Although recent advances in the treatment of CRLM have extended the possibilities to increase curability, the disease will still recur in many patients undergoing a potentially curative resection. The mean five-year survival of patients undergoing intentionally curative surgery varies from 15% to 60% [23,24,25,26,27].

Biomarkers linked to the outcome can help the management of individual patients. To this end, several prognostic biomarkers have been proposed to pilot treatment, mainly founded on clinic-pathological characteristics. Multiple prognostic factors have been identified in patients with CRLM, e.g., KRAS and BRAF mutational status, histopathological characteristics (mucinous), and surgical resection margin.

Today, computed tomography (CT) is the most widely used diagnostic tool for CRC patients, representing the first method for staging as well as a surveillance tool. In this scenario, our purpose was to assess the efficacy of radiomic features, obtained by CT examination, to predict histopathological outcomes following liver resection in colorectal liver metastases, evaluating recurrence, mutational status, histopathological characteristic (mucinous) and surgical resection margin. To the best of our knowledge, there are no studies in the literature that report radiomics analysis using both a univariate and multivariate approach, while considering linear regression models and pattern recognition techniques to predict histopathological outcomes related to the probability of developing liver metastases.

## 2. Materials and Methods

### 2.1. Dataset Characteristics

This retrospective analysis was approved by the local Ethical Committee board, and did not require informed consent from the patients due to nature of the study.

A radiological information system was accessed between January 2018 and May 2021 in order to select patients that had CRC liver metastases at staging phase, who had not been subjected to previous treatment. The inclusion criteria were (1) liver metastases with histopathological proof; (2) CT images at baseline; (3) high-quality CT images; and (4) a follow-up CT scan, taken at least six months after surgery. The exclusion criteria were (1) discordance between imaging diagnosis and the histopathological diagnosis, (2) no baseline CT images, and (3) no contrast CT images.

According to our protocol study, after liver surgery, we performed the first CT at one month, three, six months, and then every six months for the first two years in the follow-up.

The external validation patient set was obtained from Careggi Hospital, Florence, Italy.

The cohort of patients included a training set and an external validation set. The internal training set included 49 patients (18 women and 31 men) with a median age of 60 years (range 36–82 years) and 119 liver metastases. The validation cohort consisted of a total of 28 patients with single lesion (9 women and 18 men) with a median age of 61 years (range 42–78 years).

The characteristics of the patients and their metastases are summarized in Table 1.

### 2.2. CT Imaging Protocol

A 64-detector CT scanner (Optima 660, GE Healthcare, Chicago, IL, USA) set at 120 kVp and 100–470 mA (NI 16.36) was used to acquire CT images with slice thickness of 2.5 mm and table speed of 0.98–1.00 mm/rotation. The liver protocol included the same settings for each patient with unenhanced, arterial, portal, and equilibrium phases. A nonionic contrast agent (120 mL of iomeprol (Iomeron 400, Bracco, Milan, Italy)) was injected via an automatic power injector at a rate of 3 mL/s (Empower CTA, EZ-EM Inc., New York, NY, USA).

### 2.3. Image Processing

Regions of interest (ROIs) were manually segmented by two expert radiologists with 20 and 15 years of experience on liver CT, first separately, then in accordance with each other, annotating all slices of the lesions. The ROIs were segmented while avoiding distortion artifacts. Median values of features for each volume of interest were calculated. Each patient had a different number of lesions (median and range values of liver lesions are reported in Table 1).

The ROIs were delineated in the CT portal phase using the segmentation tool of 3DSlicer (https://www.slicer.org/, accessed on 16 May 2021).

### 2.4. CT Post-Processing with Pyradiomic Tool

Radiomic features were extracted by volume of interest as median values using PyRadiomics [28]. Radiomic features are subdivided into first-order statistics (19 features); shape-based (3D) (16 features); shape-based (2D) (10 features); gray-level co-occurrence matrix (24 features); gray-level run-length matrix (16 features); gray-level size-zone matrix (16 features); neighboring gray-tone difference matrix (5 features); and gray-level dependence matrix (14 features). The radiomic features comply with definitions of the imaging biomarker standardization initiative (IBSI) [29]. The descriptions are reported in (https://readthedocs.org/projects/pyradiomics/downloads/, accessed on 16 May 2021).

Radiomics analyses were performed blind to the clinical and histopathological data on baseline CT before any chemotherapy/surgical treatment. No registration techniques were applied to reduce artifacts; however, using the median value of extracted metrics, we reduced the artifacts’ influence.

### 2.5. Statistical Analysis

Statistical analysis included univariate and multivariate approaches.

#### 2.5.1. Univariate Analysis

An intraclass correlation coefficient was used to assess interobserver variability.

A nonparametric Kruskal–Wallis test was performed to identify statistically significant differences in clinical parameters and radiomic metrics between two groups (front of tumor growth: expansive versus infiltrative; tumor budding: high grade versus low grade or absent; mucinous type; and presence of recurrence).

A receiver operating characteristic (ROC) analysis was performed and the Youden index was considered to calculate the optimal cut-off value used to obtain the area under the ROC curve (AUC), sensitivity, positive predictive value (PPV), negative predictive value (NPV), and accuracy.

The statistical analyses were performed using the Statistics Toolbox of MATLAB R2007a (MathWorks, Natick, MA, USA) and a *p* value < 0.05 was considered significant.

#### 2.5.2. Multivariate Analysis

A multivariate analysis was performed in order to identify the combinations of variables that best predict the outcomes: (1) front of tumor growth: expansive versus infiltrative; (2) tumor budding: high grade versus low grade or absent; (3) mucinous type; and (4) presence of recurrence.

Given the high number of textural features, the first selection of variables was made considering only the features significant in the univariate analysis (*p* value < 0,05 at Kruskal–Wallis test) and with high accuracy considering the cut-off value, reported in Table 2.

Linear regression modeling was used to assess the best linear combination of significant textural features for each outcome. ROC analysis with the Youden index was used to identify the optimal cut-off value and to calculate AUC, accuracy, sensitivity, specificity, PPV, and NPV.

Pattern recognition methods, including support vector machine (SVM), k-nearest neighbors (KNN), artificial neural network (NNET), and decision tree (DT), were made to assess the performance in a multivariate procedure [30]. The best multivariate model was chosen considering the highest accuracy. Training was performed using 10 k-fold cross-validation. Moreover, an external validation cohort was used to validate the findings of the best classifier. Machine Learning Toolbox of MATLAB R2007a (MathWorks, Natick, MA, USA) was used.

## 3. Results

### 3.1. Univariate Analysis Findings

The median value of intraclass correlation coefficients for features was 0.92 (range 0.87–0.96). The lesion size did not affect the extracted metrics (*p*-value > 0.05 at the Kruskal–Wallis test performed between the groups: patients with lesions < 3.6 cm and patients with lesions ≥ 3.6 cm; the median size of lesions in our population). In addition, the RAS mutational status did not affect the extracted metrics (*p*-value > 0.05 at the Kruskal–Wallis test performed between the groups; therefore, considering the two groups homogeneous regarding the extracted radiomic metrics, RAS mutational status was not considered for the following analysis).

Among significant features that differentiate the front of tumor growth, seven textural parameters obtained an accuracy ≥75%. Among these seven features, the best performance in discriminating expansive versus infiltrative front of tumor growth was wavelet_HHL_glcm_Imc2 with an accuracy of 79%, a sensitivity of 84%, a specificity of 67%, and a PPV and NPV of 83% and 69%, respectively, with a cut-off value of 0.13 (Table 3).

Among significant features that differentiate the tumor budding, 16 textural parameters obtained an accuracy ≥80%. Among these 16 features, the best performance in discriminating expansive versus tumor budding was wavelet_LLL_firstorder_Mean with an accuracy of 86%, a sensitivity of 91%, a specificity of 65%, and a PPV and NPV of 90% and 68%, respectively, with a cut-off value of 215.32 (Table 3).

Among the significant features differentiating the mucinous type of tumor, 15 textural parameters obtained an accuracy ≥80%. Among these 15 features, the best performance in differentiating the mucinous type of the tumor was original_firstorder_RobustMeanAbsoluteDeviation with an accuracy of 88%, a sensitivity of 42%, a specificity of 100%, and a PPV and NPV of 100% and 86%, respectively, with a cut-off value of 20.34 (Table 3).

Among significant features that identify tumor recurrence on portal phase, 16 textural parameters obtained an accuracy ≥80%. Among these 16 features, the best performance in identifying tumor recurrence was wavelet_HLH_glcm_Idmn with an accuracy of 85%, a sensitivity of 81%, a specificity of 88%, and a PPV and NPV of 78% and 89%, respectively, with a cut-off value of 0.99 (Table 3).

### 3.2. Multivariate Analysis Findings

#### 3.2.1. Linear Regression Analysis Findings

**Figure 1:**
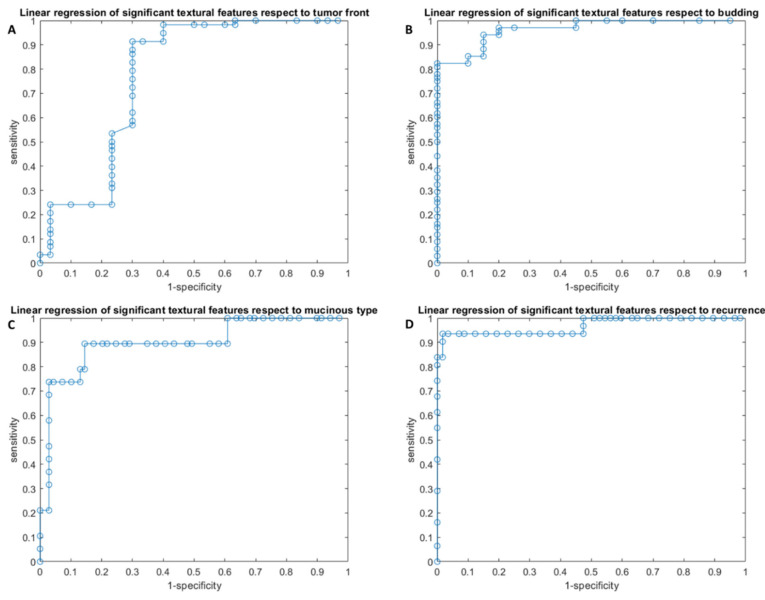
ROC curves of linear regression analysis with respect to the front of tumor growth (**A**), tumor budding (**B**), tumor mucinous type (**C**), and recurrence presence (**D**) obtained considering significant features.

Linear regression models obtained good results, with accuracy of 84–97% (Table 4, Figure 1), in each considered classification problem: (1) Front of tumor growth: expansive versus infiltrative; (2) tumor budding: high grade versus low grade or absent; (3) mucinous type; and (4) presence of recurrence. The best linear regression model was obtained in the identification of recurrence considering the linear combination of the 16 significant textural metrics extracted by the CT portal phase (AUC of 0.95, accuracy of 97%, sensitivity of 94%, and specificity of 98%). 

The linear combination of seven significant textural features in the detection of tumor front growth reached an accuracy of 84%, with a sensitivity of 91% and a specificity of 70%; the linear combination of the 16 significant textural parameters in the detection of tumor budding reached an accuracy of 86%, with a sensitivity of 82% and a specificity of 100%. The linear regression model of 15 significant textural metrics to differentiate mucinous tumor obtained an accuracy of 86%, with a sensitivity of 89% and a specificity of 86%.

The coefficients of these linear models are reported in Table 5.

#### 3.2.2. Pattern Recognition Approaches Findings

Considering significant textural metrics tested with pattern recognition approaches, the best performance for each outcome ((1) front of tumor growth: expansive versus infiltrative; (2) tumor budding: high grade versus low grade or absent; (3) mucinous type; and (4) presence of recurrence) was reached using KNN as a classifier considering the significant features extracted by the CT portal phase.

The accuracy for each classification problem was greater than 86% (Table 4) in the training and validation sets, and the best results were obtained in the identification of tumor front growth with the seven significant textural features (AUC of 0.95, an accuracy of 97%, sensitivity of 90%, and a specificity of 100%).

**Figure 2:**
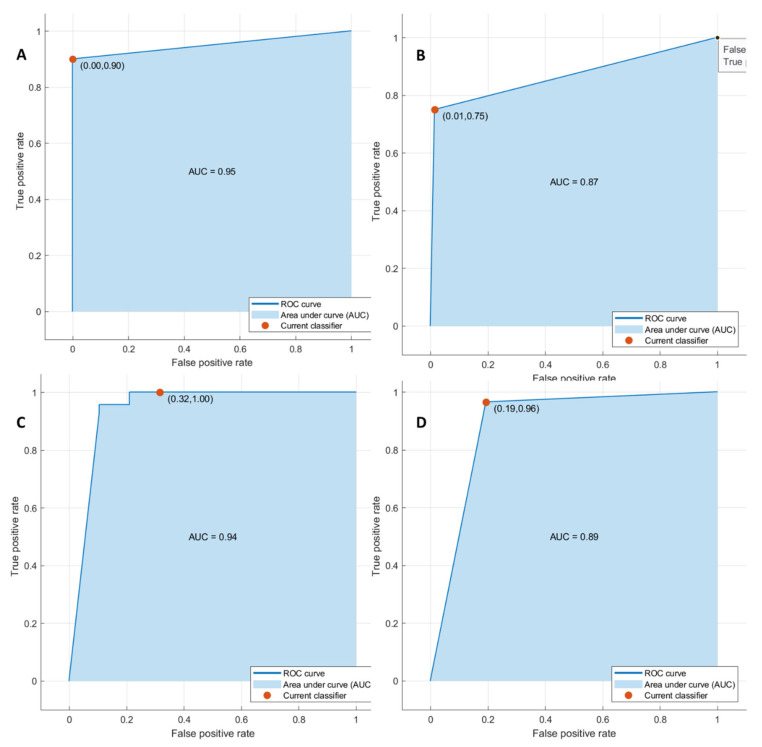
ROC curves of KNN with respect to the front of tumor growth (**A**), tumor budding (**B**), tumor mucinous type (**C**), and recurrence presence (**D**) obtained considering significant features in internal training set.

**Figure 3:**
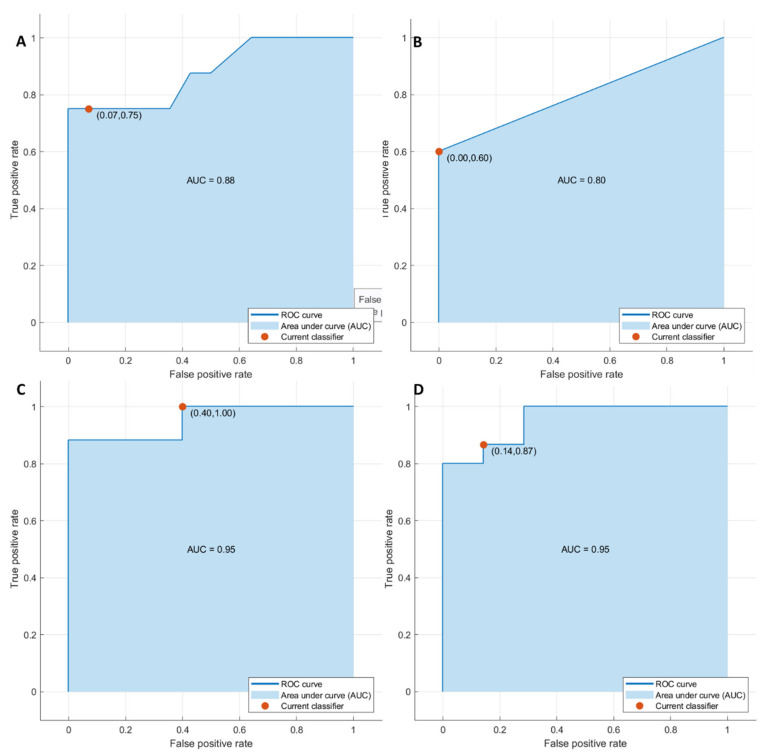
ROC curves of KNN with respect to the front of tumor growth (**A**), tumor budding (**B**), tumor mucinous type (**C**), and recurrence presence (**D**) obtained considering significant features in external validation test.

The KNN of 16 significant textural features in the detection of tumor budding reached an accuracy of 93%, with a sensitivity of 75% and a specificity of 99%; the KNN of the 15 significant textural parameters in the differentiation of mucinous type reached an accuracy of 93%, with a sensitivity of 100% and a specificity of 68%. The KNN classifier of 16 significant textural metrics to detect recurrences obtained an accuracy of 91% with a sensitivity of 96% and a specificity of 81% (Figure 2 and Figure 3).

## 4. Discussion

Several studies evaluating radiomics and radiogenomics data in CRLM patients pointed out their use in early detection, treatment assessment, and prognosis. Regarding the prognosis, the assessment and prediction of response to systemic neoadjuvant treatment or liver resection are crucial in preventing a delay in the choice of alternative therapies. Additionally, in patients unfit for surgery, predicting the response to therapy may prevent unsuccessful treatment regimens and major side effects.

Several studies showed that low skewness was associated with a high response rate to chemotherapy with FOLFOX or FOLFIRI; these data were validated in an external cohort [31]. Giannini et al. showed that heterogeneity features were related to dual anti-Her2 treatment response [32]. Several studies showed that high entropy and low homogeneity were related to earlier response, showing an association between entropy and prognosis [33,34,35,36,37,38]. Andersen et al. showed a correlation between homogeneity features and worse overall survival (OS) [34]. However, Rahmim et al. showed heterogeneity obtained by FDG PET was related to lower OS [39].

Lubner et al. demonstrated that the degree of skewness was inversely correlated to KRAS status, and entropy with OS [36]. In addition to the survival benefits of several data, the possibility of stratifying patients for recurrence in liver has been demonstrated [37,38,39,40,41]. Ravanelli et al. correlated high uniformity and low OS and PFS in CRLM patients [41].

According to Simpson et al. [37], we obtained good performance considering the single textural significant metric in the identification of the front of tumor growth (expansive versus infiltrative) and tumor budding (high grade versus low grade or absent), in the recognition of mucinous type, and in the detection of recurrences. At univariate analysis, the best performance in discriminating expansive versus infiltrative front of tumor growth was wavelet_HHL_glcm_Imc2 with an accuracy of 79%, a sensitivity of 84%, and a specificity of 67%. The best performance in discriminating expansive versus tumor budding was wavelet_LLL_firstorder_Mean with an accuracy of 86%, a sensitivity of 91%, and a specificity of 65%. The best performance in differentiating the mucinous type of the tumor was original_firstorder_RobustMeanAbsoluteDeviation with an accuracy of 88%, a sensitivity of 42%, and a specificity of 100%. The best performance in identifying tumor recurrence was wavelet_HLH_glcm_Idmn with an accuracy of 85%, a sensitivity of 81%, and a specificity of 88%. However, considering a linear regression model or a neural network classifier in a multivariate approach, it was possible to increase the performance. The best linear regression model was obtained with the identification of recurrence considering the linear combination of the 16 significant textural metrics extracted by CT portal phase, which reached an AUC of 0.95, an accuracy of 97%, a sensitivity of 94%, and a specificity of 98%. The best results with KNN were obtained with the identification of tumor front growth with the seven significant textural features, which reached an AUC of 0.95, an accuracy of 97%, a sensitivity of 90%, and a specificity of 100%. Computer-based image analysis, such as texture analysis, has the potential to detect changes in liver parenchymal enhancement. TA quantifies heterogeneity at the pixel level in CT images. The texture features of liver parenchyma may be altered by occult malignancy and may represent a potential surrogate for later recurrent disease [37]. Unlike Simpson et al. [37], our data were not influenced by any type of treatment, as we evaluated native patients. Our results confirmed the capacity of radiomics data to identify several prognostic features that may affect the treatment choice in patients with liver metastases, in order to obtain a more personalized approach and avoid unnecessary treatments [42,43,44,45,46,47,48,49,50]. The ability to obtain a prognostic biomarker allows the multidisciplinary team to correctly manage the patient.

These results were confirmed by an external validation dataset. Nevertheless, several limits of radiomics analysis cause difficult standardization in clinical settings. The main limit is related to the different software evaluations in distinct investigations, together with the variety of imaging devices in different diagnostic centers. Another limit is the lesion segmentation, which may affect results.

The present study has several limits: the small population size evaluated; the retrospective nature of the study; and the manual segmentation that, in our opinion, is more realistic, despite several studies supporting automatic segmentation to avoid inter-observer variability. Moreover, we did not assess the impact of CT contrast administration and the different phases of contrast study. Disease recurrence should be the topic of a future paper with an adequate validation cohort.

## 5. Conclusions

We obtained good performance considering the single significant textural metric in the identification of the front of tumor growth (expansive versus infiltrative) and tumor budding (high grade versus low grade or absent), in the recognition of mucinous type, and in the detection of recurrences. Additionally, considering a linear regression model or a neural network classifier in a multivariate approach, it was possible to increase the performance. The best linear regression model was obtained with the identification of recurrence considering the linear combination of the 16 significant textural metrics extracted by the CT portal phase, which reached an AUC of 0.95, an accuracy of 97%, a sensitivity of 94%, and a specificity of 98%. The best results with a KNN were obtained with the identification of tumor front growth considering seven significant textural features, which reached an AUC of 0.95, an accuracy of 97%, a sensitivity of 90%, and a specificity of 100%. Our results confirmed the capacity of radiomics data to identify several prognostic features that may affect the treatment choice in patients with liver metastases, in order to obtain a more personalized approach. These results were confirmed by external validation dataset.

## Figures and Tables

**Table 1:** Characteristics of the study population (77 patients).

Patient Description	Numbers (%)/Range
Sex	Men 50 (64.9%)
	Women 27 (35.1%)
Age	61 year; range: 36–82 year
Primary cancer site	
Colon	52 (67.5%)
Rectum	25 (32.5%)
Hepatic metastases description	
Patients with single nodule	48 (62.3%)
Patients with multiple nodules	29 (37.7%)/range: 2–13 metastases
Nodule size (mm)	median size 35.8 mm; range 7–58 mm
Front of tumor growth	
Expansive	28 (36.4%)
Infiltrative	49 (63.6%)
Tumor budding	
Absent	11 (14.3%)
Low grade	13 (16.9%)
High grade	53 (68.8%)
Mucinous carcinoma	25 (32.5%)
Recurrence presence	19 (24.7%)
RAS mutational status	39 (50.6%)

**Table 2:** (Sub) datasets, variable selection criteria, and predictor combinations.

	Outcome Variable	Predictors	Accuracy Threshold on Univariate Analysis
Dataset 1	Front of tumor growth	Significant radiomic metrics on lesion by univariate analysis	≥0.75
Dataset 2	Tumor budding		≥0.80
Dataset 3	Mucinous type		≥0.80
Dataset 4	Recurrence presence		≥0.80

**Table 3:** Findings by univariate analysis with ROC performance results.

Best Predictor at Univariate Analysis	Respect to Tumor Growth Front	Respect to Tumor Budding	Respect to Mucinous Type	Respect to Recurrences
	wavelet_HHL_glcm_Imc2	wavelet_LLL_firstorder_Mean	original_firstorder_RobustMeanAbsoluteDeviation	wavelet_HLH_glcm_Idmn
AUC	0.73	0.73	0.62	0.8
Sensitivity	0.84	0.91	0.42	0.81
Specificity	0.67	0.65	1.00	0.88
PPV	0.83	0.9	1.00	0.78
NPV	0.69	0.68	0.86	0.89
Accuracy	0.79	0.86	0.88	0.85
Cut-off	0.13	215.32	20.34	0.99

**Table 4:** Linear regression and pattern recognition analysis with significant features from the portal phase.

Linear Regression of Significant Features	AUC	Sensitivity	Specificity	PPV	NPV	Accuracy	Cut-Off
Linear regression of the textural features with respect to the lesion front	0.74	0.91	0.70	0.85	0.81	0.84	1.51
Linear regression of the textural features with respect to tumor budding	0.91	0.82	1.00	1.00	0.63	0.86	1.43
Linear regression of the textural features with respect to the mucinous type	0.87	0.89	0.86	0.63	0.97	0.86	0.31
Linear regression of the textural features with respect to recurrence	0.95	0.94	0.98	0.97	0.97	0.97	0.44
Pattern recognition analysis with significant features	Dataset	AUC with 95% of confidence interval	Accuracy	Sensitivity	Specificity	Training time [sec]	Model type and parameters
KNN	Training set	0.95 (0.92–0.97)	96.60	90.00	100.00	8.70	Weighted KNN; number of neighbors: 10; distance metric: Euclidean; distance weight: squared inverse
	Validation set	0.88 (0.85–0.90)	86.40	75.00	93.00		
	Training set	0.87 (0.82–0.91)	93.20	75.00	99.00	8.90	
	Validation set	0.80 (0.78–0.84)	90.90	75.00	100.00		
	Training set	0.94 (0.92–0.97)	93.20	100.00	68.00	9.10	
	Validation set	0.95 (0.91–0.96)	90.90	100.00	60.00		
	Training set	0.89 (0.85–0.92)	90.90	96.00	81.00	7.80	
	Validation set	0.95 (0.91–0.98)	86.40	87.00	86.00		

**Table 5:** Linear regression model parameters.

Linear regression of the textural features with respect to the front of tumor growth	Coefficients	*p*-value	*p*-value
Intercept	0.34	0.81	0.00
original_firstorder_RootMeanSquared	0.00	0.12	
wavelet_LLH_firstorder_Skewness	0.09	0.00	
wavelet_HHH_glcm_JointEntropy	2.87	0.06	
wavelet_HHH_glcm_Imc2	−2.23	0.10	
wavelet_HHH_glcm_Imc2	−9.22	0.12	
wavelet_HHL_glcm_MCC	−0.70	0.16	
wavelet_HHL_glcm_Imc2	2.54	0.07	
Linear regression of the textural features with respect to tumor budding	Coefficients	*p*-value	*p*-value
Intercept	−0.21	0.86	0.00
original_firstorder_Median	0.20	0.04	
original_firstorder_RootMeanSquared	0.58	0.00	
original_firstorder_10Percentile	0.09	0.03	
original_firstorder_Mean	−0.82	0.00	
wavelet_LHL_glcm_Imc2	6.70	0.00	
wavelet_LLH_glcm_ClusterShade	0.00	0.58	
wavelet_HHH_gldm_SmallDependenceHighGrayLevelEmphasis	−0.20	0.01	
wavelet_HHL_glcm_MCC	−0.55	0.54	
wavelet_HHL_glcm_Imc2	4.69	0.04	
wavelet_HHL_ngtdm_Strength	−5.58	0.22	
wavelet_LLL_firstorder_Uniformity	34.99	0.08	
wavelet_LLL_firstorder_Median	−0.04	0.25	
wavelet_LLL_firstorder_RootMeanSquared	−0.22	0.00	
wavelet_LLL_firstorder_10Percentile	−0.03	0.04	
wavelet_LLL_firstorder_Mean	0.27	0.00	
wavelet_LLL_glrlm_GrayLevelNonUniformityNormalized	−44.55	0.06	
Linear regression of the textural features respect to the mucinous type	Coefficients	*p*-value	*p*-value
Intercept	0.35	0.42	0.00
original_firstorder_Median	−0.02	0.75	
original_firstorder_RobustMeanAbsoluteDeviation	0.02	0.46	
original_firstorder_RootMeanSquared	0.15	0.07	
original_firstorder_10Percentile	0.01	0.80	
original_firstorder_Mean	0.00	1.00	
wavelet_LLH_glcm_Imc2	0.33	0.52	
wavelet_LLH_ngtdm_Strength	−0.03	0.48	
wavelet_HLH_ngtdm_Strength	−1.13	0.29	
wavelet_HLH_ngtdm_Busyness	0.00	0.02	
wavelet_HHH_glcm_Imc2	0.69	0.60	
wavelet_HHH_ngtdm_Strength	3.47	0.14	
wavelet_LLL_firstorder_Median	0.01	0.75	
wavelet_LLL_firstorder_RootMeanSquared	−0.06	0.07	
wavelet_LLL_firstorder_10Percentile	0.00	0.99	
wavelet_LLL_firstorder_Mean	0.00	0.95	
Linear regression of the textural features with respect to recurrences	Coefficients	*p*-value	*p*-value
Intercept	−0.70	0.77	0.00
wavelet_LHH_gldm_DependenceVariance	0.01	0.59	
wavelet_HLH_gldm_LargeDependenceHighGrayLevelEmphasis	0.00	0.01	
wavelet_HLH_glcm_ClusterShade	−1.60	0.02	
wavelet_HLH_glcm_Idmn	−26.27	0.00	
wavelet_HLH_glcm_Idn	29.13	0.00	
wavelet_HLH_glcm_ClusterProminence	0.06	0.04	
wavelet_HLH_firstorder_Skewness	0.29	0.01	
wavelet_HLH_firstorder_Maximum	0.00	0.17	
wavelet_HLH_firstorder_Range	−0.01	0.01	
wavelet_HLH_firstorder_Kurtosis	0.01	0.01	
wavelet_HLH_glrlm_LongRunHighGrayLevelEmphasis	0.01	0.17	
wavelet_HLH_glszm_GrayLevelVariance	0.14	0.13	
wavelet_HLH_glszm_HighGrayLevelZoneEmphasis	0.04	0.00	
wavelet_HLH_ngtdm_Complexity	0.01	0.34	
wavelet_LLL_glszm_SizeZoneNonUniformityNormalized	5.74	0.03	
wavelet_LLL_glszm_SmallAreaEmphasis	−2.23	0.30	

## Data Availability

Data is available at the following link: https://zenodo.org/record/6372793#.YjoXzOrMK3A.
